# Supplementary material for: Evaluation of NEUROG1 methylation status in stool specimens in the advanced adenomas and colorectal cancer
Source: PeerJ. 2025 Oct 8;13:e19968. doi: 10.7717/peerj.19968 (PMC12514994; doi:10.7717/peerj.19968)
Supplement: Supplemental Information 3 [file peerj-13-19968-s003.docx]

GENDER: 1=MALE 2=FEMALE

MARKER3: NEUROG1

ACTB: ACTIN BETA

M3AY= $7.867-0.097*NEUROG1-0.098*ACTB$

M3AE=

$$\frac{1}{1+e^{-(7.867-0.097*NEUROG1-0.098*ACTB)}}$$

SPSS software (IBM Corp., Armonk, NY, USA) was used to conduct the fitting logistic regression analysis of CP values for target genes and internal reference genes, and the fitting formula was obtained:

$$\frac{1}{1+e^{-(7.867-0.097*NEUROG1-0.098*ACTB)}}$$

, in which the sample is positive if the fitting value was >0.7692
